# Dynamics of DNA methylation during osteogenic differentiation of porcine synovial membrane mesenchymal stem cells from two metabolically distinct breeds

**DOI:** 10.1080/15592294.2024.2375011

**Published:** 2024-07-02

**Authors:** Shuaichen Li, Puntita Siengdee, Frieder Hadlich, Nares Trakooljul, Michael Oster, Henry Reyer, Klaus Wimmers, Siriluck Ponsuksili

**Affiliations:** a Institute of Genome Biology, Research Institute for Farm Animal Biology (FBN), Dummerstorf, Germany; b Program in Applied Biological Sciences: Environmental Health, Chulabhorn Graduate Institute, 906 Kamphaeng Phet 6 Road, Lak-Si, Bangkok, Thailand; c Faculty of Agricultural and Environmental Sciences, University of Rostock, Rostock, Germany

**Keywords:** DNA methylation, Mesenchymal stem cells, Osteogenic differentiation, Pig breeds, Epigenetic pattern

## Abstract

Mesenchymal stem cells (MSCs), with the ability to differentiate into osteoblasts, adipocytes, or chondrocytes, show evidence that the donor cell’s metabolic type influences the osteogenic process. Limited knowledge exists on DNA methylation changes during osteogenic differentiation and the impact of diverse donor genetic backgrounds on MSC differentiation. In this study, synovial membrane mesenchymal stem cells (SMSCs) from two pig breeds (Angeln Saddleback, AS; German Landrace, DL) with distinct metabolic phenotypes were isolated, and the methylation pattern of SMSCs during osteogenic induction was investigated. Results showed that most differentially methylated regions (DMRs) were hypomethylated in osteogenic-induced SMSC group. These DMRs were enriched with genes of different osteogenic signalling pathways at different time points including Wnt, ECM, TGFB and BMP signalling pathways. AS pigs consistently exhibited a higher number of hypermethylated DMRs than DL pigs, particularly during the peak of osteogenesis (day 21). Predicting transcription factor motifs in regions of DMRs linked to osteogenic processes and donor breeds revealed influential motifs, including *KLF1, NFATC3, ZNF148, ASCL1, FOXI1*, and *KLF5*. These findings contribute to understanding the pattern of methylation changes promoting osteogenic differentiation, emphasizing the substantial role of donor the metabolic type and epigenetic memory of different donors on SMSC differentiation.

## Introduction

Mesenchymal stem cells (MSCs) are multipotent cells with the ability to differentiate into various cell types of the mesodermal lineage, which have been widely used in regenerative medicine. They were first isolated from the bone marrow and then had been found in various other tissues, while the ability of these MSCs to differentiate into different cell types varies according to tissue source. Despite their robust osteogenic ability, MSCs derived from bone marrow are rare and always need invasive procedures. Studies have reported that synovial membrane mesenchymal stem cells (SMSCs) have better osteogenic potential compared with MSCs derived from adipose [1,2]. Therefore, SMSCs have generated great interest in the treatment of bone and joint diseases [3]. We have previously characterized the porcine SMSCs and investigated the differential gene expression patterns during in vitro osteogenic induction [4]. However, the underlying epigenetic patterns that regulate osteogenesis of SMSCs are as yet poorly understood.

Epigenetics refers to no alteration in DNA sequence itself, but to mitotically and meiotically heritable changes in gene expression. DNA methylation on the fifth position of cytosine (5mC) is one of the main epigenetic modifications which predominantly happens as paired symmetric methylation of CpG dinucleotide. DNA methyltransferases (DNMTs), including DNMT1, DNMT3a, and DNMT3b, are responsible for catalysing the methylation of CpG dinucleotides. DNMT1 functions as a maintenance methyltransferase, preserving existing methylation patterns, while DNMT3a and DNMT3b serve as de novo methyltransferases, establishing new methylation marks [5]. Many studies have suggested that DNA methylation is required for osteogenic differentiation [6,7]. It has been observed that the supplement of DNA demethylating agent leads to increased osteogenic performance of MSCs [8,9]. DNA methylation can directly regulate transcription by interfering with the recognition sites of transcription factors (TFs) [10]. Meanwhile, several key TFs, such as RUNX2, PPAR γ2, and SOX9, are thought to regulate osteogenic differentiation of MSCs by DNA methylation [11–13]. Although it is commonly perceived that DNA methylation dynamically regulates diverse biological processes [14], only a few studies have described the global DNA methylation changes in osteogenic lineage commitment [15–17]. More importantly, little attention has been paid to the temporal modulation of genome-scale DNA methylation at the single nucleotide level during osteogenic differentiation. The dynamic characterization of DNA methylation changes can contribute to a better understanding of osteogenic differentiation and bone development.

There is mounting evidence of the complex interplay between intermediary metabolism in bone and whole-body metabolism [18]. There is increasing evidence that the metabolic type of the donor cell plays an important role in the osteogenic process of the mesenchymal stem cells. There has been evidence that the osteogenic capacity of MSCs from donors with obesity and/or metabolic disorders is impaired [19–22]. Pigs are seen as an attractive option for bone and MSCs research [23,24], while recent studies explored the epigenetic differences between MSCs isolated from lean and obese pigs [25–27]. Although these studies showed how extrinsic dietary factors affect the epigenetic landscape at the DNA and histone level, it remains unclear whether the intrinsic epigenetic differences of pig breeds with distinct metabolic patterns can influence the molecular characteristics of MSCs, especially in the process of osteogenic differentiation. Traditional pig breeds, like Angeln Saddleback (AS), tend to have higher fat content compared to modern breeds, such as German Landrace (DL). Understanding the epigenetic differences of MSCs derived from donors with distinct inheritable metabolic traits in osteogenic differentiation will allow to optimization of therapeutic osteogenic applications.

Therefore, in this study, we investigated the genome-wide DNA methylation changes of pig SMSCs during in vitro osteogenic differentiation, taking into account not only different time points but also two pig breeds with different metabolic features. We also performed an integrative analysis on DNA methylation and gene expression data from our previous study [4], as well as transcription factor-binding motifs enrichment analysis, in order to identify latent methylation-driven genes and key transcription factor during in vitro osteogenesis.

## Materials and Methods

### Isolation, culture and osteogenic differentiation of porcine SMCSs

We used cell samples from our previous study, which were derived from fibrous synovial tissue of the knee joint of piglets of two breeds: German Landrace (DL, *n* = 3) and Angeln Saddleback (AS, *n* = 3) [4]. These SMSCs expressed stemness markers CD44, CD29, CD90, and CD105, confirmed by flow cytometry, and could differentiate into osteocytes, adipocytes, and chondrocytes [28]. The isolation methods, culture conditions, morphological aspects, and molecular characterization of synovial mesenchymal stem cells (SMSCs) were detailed in our previous study [4,28]. Our previous study demonstrated that during osteogenic differentiation, mineral deposition was first observed at day 14 and further increased until day 21 as confirmed by the osteogenic markers including *ALPL, COL1A1, RUNX2* and *NANOG* [4]. In brief, after SMSCs at passage 3 became nearly 80% of confluence, cells for osteogenic induction (Ost) were incubated with osteogenic differentiation medium (ODM), while the control undifferentiated (Con) SMSCs remained in complete culture medium (CCM) supplemented with 10% FBS and 1% antibiotic/antimycotic solution. On culturing days (D) 0, 1, 7, 14, and 21, cell samples from each breeds and groups were collected, then pooled across individuals for RNA and DNA extraction. A total of 36 samples were used: differentiating cells (Ost): non-differentiated cells (Con): collected at 5 time points (D 0, 1, 7, 14, 21) × 2 breeds × 2 technical replicates; collected at 4 time points (D 1, 7, 14, 21) × 2 breeds × 2 technical replicates.

## Gene expression analysis of DNA methyltransferases (DNMTs) by Quantitative PCR

As previously described, RNAs were extracted by TRI reagent (Sigma-Aldrich) and reverse transcribed using SuperScript II (Invitrogen) and Oligo (dT). The geometric mean of three reference genes (*HPRT1*, *PPIA*, and *YWHAZ*) was used for normalization. The primers sequence was shown in Supplementary Table S1. The statistical analysis was performed using the MIXED procedure in SAS v. 9.4 (SAS Institute Inc.) to analyse the differential gene expression between breeds and the state of SMSCs (states_days) and their interactions with breed. The adjusting for multiple comparisons were done by using the post hoc Tukey–Kramer test.

## DNA extraction, library construction, and RRBS

Genomic DNA was isolated using the DNeasy kit (Qiagen). For library preparation, 2 μg of DNA with a 1% spike-in control (un-methylated cl857 Sam7 Lambda DNA, Promega) digested with double enzyme (MspI and TaqI). Fragments were then end-repaired, A-tailed and ligated to c-methylated adapters using a TruSeq Nano DNA Sample Preparation kit (Illumina) according to the manufacturer’s recommendations. The Illumina TruSeq DNA library preparation kit was used for multiplexing samples per sequencing lane. After this, 2% low-range ultra-agarose gels were used to obtain proper sized adapter-ligated DNA fragments (40–240 bp). Bisulphite conversion was performed with the EpiTect Bisulphite kit (Qiagen). The bisulphite-converted libraries were then PCR amplified using a PfuTurbo Cx Hotstart DNA Polymerase kit (Stratagene). The quality of RRBS libraries was assessed using an Agilent DNA 1000 kit (Agilent Technologies). Next-generation sequencing of the RRBS libraries was performed on an Illumina HiSeq2500 at the FBN, Dummerstorf, Germany.

As previously reported, a standard analysis pipeline of RRBS sequencing has been established by our group including base calling and alignment. Briefly, in the raw fastq files, sequence reads with a mean Phred quality (Q-score) > 20, a minimum length of 30 bp without uncertain base-calling of N and adapter sequence contamination were retained. After a 2bp-trimming both 5’ and 3’ fragment ends, the clean reads were mapped to the reference genome (Sscrofa 11.1) using Bowtie2 version 2.2.8. Subsequently, read coverages and methylation percentages of each cytosine were obtained by Bismark version 0.19.0. In this study, only cytosines in CpG context were considered for further analysis, while non-CpG (CHG and CHH where H is A, C, or T) sites were discarded. In addition, to filter out SNPs which can cause bias in methylation call, we also sequenced non-bisulphite-treated version of reduced representation DNA libraries according to previously described procedure [29]. CpG sites overlapping with SNPs were filtered out.

## Differentially methylation analysis and annotation

Since CpG methylation is symmetric, read counts from both strands were aggregated to form a single value for further analysis. The CpG sites with less than 2 coverage or more than the 99.9th percentile of coverage in each sample were omitted. The R package DSS (v2.48.0) was used to identify differential methylation loci (DML) and differential methylation regions (DMR). DSS-general implements a statistical method to analyse bisulphite sequencing data from experimental designs, which fits a beta-binomial generalized linear model with an arcsine link function and Wald tests [30]. In this study, the differentiation states (Ost, Con) and time-point of cell collection were considered as a combined factor (factor states_days; 9 levels: ConD0, ConD1, ConD7, ConD14, ConD21, OstD1, OstD7, OstD14, OstD21). A full model, including states_days, breeds (factor breed; 2 levels: AS, DL) and their interaction was fitted using DMLfit.multiFactor function with a smoothing algorithm. Subsequently, the contrast matrixes for comparisons of interest were created and passed to DMLtest.multiFactor function [31]. After obtaining DMLs, the callDMR function was used to get DMRs (parameters: p.threshold = 0.05). The areaStat values provided by DSS were used to rank significant DMRs and determine the direction of methylation (hyper- or hypomethylation). The minimal absolute value of areaStat was 11 in this study. The methylation levels of each DMRs were defined as weighted average of the methylation levels of CpGs located in the DMRs. The weighting factors were the coverage data of CpGs.

The annotation files of NCBI RefSeq (including 77,708 transcripts) and CpG Islands (CGIs) from genome assembly Sscrofa11.1 were downloaded from the UCSC Table Browser. All CpG sites and DMRs annotation to gene features were performed using ChIPseeker package (v3.17) [32]. For genomic features, the promoter regions were defined as ±2 kb from the Transcription Start Site (TSS). For CpG annotation, CGI shore and CGI shelf were defined as 2 kb regions flanking CGIs and 2 kb regions flanking CGI shore, respectively.

The VennDiagram package (v1.7.3) was used to draw the intersection of gene symbols of annotated DMRs from each comparisons. The hyper- and hypomethylated DMRs were plotted against the number of CpGs located in DMRs from each comparisons by EnhancedVolcano package (v1.18.0). We further calculated the mean methylation levels of each DMRs in the same groups from different comparisons. The DMRs with a relative methylation difference ≥ 30% between any of comparisons were considered for visualization by ComplexHeatmap package (v2.16.0) [33].

## Functional enrichment analysis

DMRs from each comparisons were uploaded to the Database for Annotation Visualization and Integrated Discovery (DAVID) for functional and pathway enrichment analysis. The biological process (BP) enrichment and KEGG pathway were performed with default setting. Only terms with *P*-value <0.05 and count > 4 were considered. In addition, terms related to cancer and infection will not be discussed in this study.

## Longitudinal pattern recognition

To characterize DNA methylation changing over time, we made use of Short Time-Series Expression Miner (STEM) algorithm to cluster DMRs [34]. The methylation data of each DMRs in SMSCs samples prior to (D0) and after osteogenic induction (D1, 7, 14, and 21) were fed into STEM (v1.3.12) and run by default. The median methylation level of the osteogenic induction from days 1, 7, 14 and 21 were normalized to day 0 data, which was used as the reference. The gene symbols of each significant profile (*P*-value <0.05) were submitted to IPA for canonical pathways analysis.

## Gene expression profiling

The gene expression data of same samples used in methylation data were based on the Affymetrix porcine snowball array (SNOWBALLs520824F). The expression analysis was performed using a subset of data from previously published dataset (GEO accession number: GSE219289) [4]. To explore the relationship between DMRs and its related genes, we used Hmisc package (v5.1–1) to calculate Pearson correlation coefficients (r) and significance levels (*p-*value). The potential DNA methylation driven genes with negative correlation and *p-*value *< 0.05* were shown.

## Transcription factor binding site (TFBS) at DMRs

To explore the association between TFBS and DNA methylation during osteogenic differentiation, we selected promoter DMRs to determine if experimentally defined TFBS are enriched in these DMRs. The TFBS profiles were retrieved from JASPAR2022 CORE non-redundant database [35]. We used the TFBSTools package (v1.38.0) to recognize putative TFBS in these DMRs [36]. The runAme function from memes package (v1.8.0) was performed in the ‘Vs Shuffled’ mode for TFBS motif enrichment [37]. To further narrow down the result, only TFBS included in DMRs with larger than 30% of average methylation differences between any of comparisons were presented.

## Results

### DNMTs expression profiles and global DNA methylation during osteogenic induction of SMSCs

**Figure 1.**
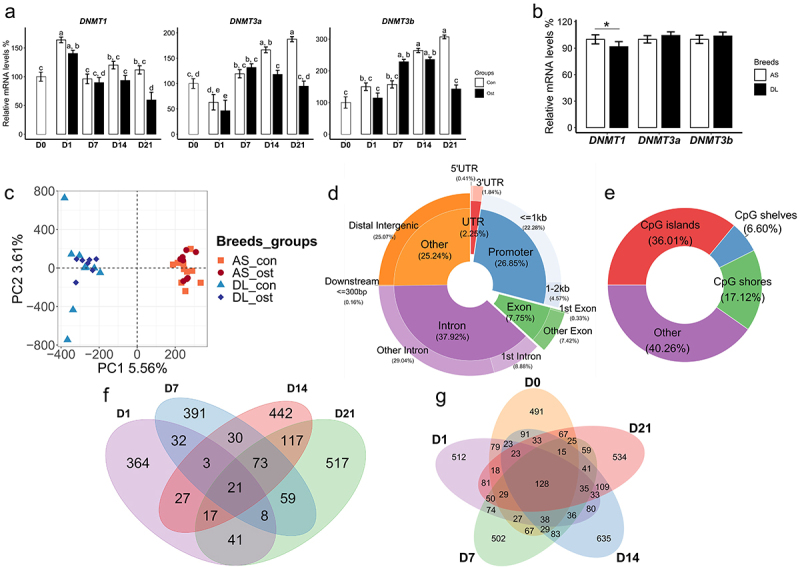
(a) Gene expression level of three DNMTs of SMSCs grown in growth medium (Con) and osteogenic medium (Ost) over 21 days. (b) Gene expression level of three DNMTs of SMSCs derived from as and DL pigs. (c) Genic annotation of CpG sites. (d) Genomic annotation of CpG sites to DNA-methylation environments. (e) Principal component analysis (PCA) of methylation data of CpG sites. (f) Venn diagram showing unique and overlapping DMR-associated genes between control SMSCs and osteogenic induced SMSCs at each time point. (g) Venn diagram showing unique and overlapping DMR-associated genes between as and DL prior to and after osteogenic differentiation at each time point.

In the comparisons of Ost group and Con group, besides a reduced expression of *DNMT3a* on day 14, the expressions of all three DNMTs (*DNMT1, DNMT3a*, and *DNMT3b*) were significantly downregulated in osteogenic-induced SMSCs on day 21 (Figure 1a). We also found that SMSCs from AS pigs have higher expression of *DNMT1* compared to those from DL pigs, which corresponds to higher DNA methylation in AS (Figure 1b).

In total, we obtained 1 billion sequence reads for all samples with an average mapping efficiency of 53.3% (Suplementary Table S2). After quality check and filtering steps, around 2.78 million CpGs were detected in all samples. For genomic annotations, about 45.67% of these CpGs were mapped to the gene body region (introns and exons), while CpGs in the promoter and distal intergenic regions account for 26.85% and 25.07%, respectively (Figure 1c). For CpG annotations, the share of CpGs located in CpG islands was 36.01%, while CpGs in the CpG shores and shelves region account for 17.12% and 6.6%, respectively (Figure 1d). Principal component analysis (PCA) of all CpGs showed that sample segregation was more distinct between pig breeds (AS vs DL) than between SMSCs groups (Ost vs Con) for the first two principal components (Figure 1e). At the global level, median methylation rates were similar between the Ost and Con groups at all-time points, while SMSCs from AS tended to have higher median methylation rates than those from DL. In addition, promoter and CpG islands regions were uniformly hypomethylated across all samples.

## Identification of DMRs in response to osteogenic induction

The numbers of DMLs (FDR <0.1) between osteogenic and control SMSCs were 18, 298, 622, and 992 from day 1 to day 21. For the comparisons between AS and DL, we found 888, 937, 1150, 1456, and 1062 DMLs (FDR <0.1) between day 0 and day 21. In view of the fact that methylation at several proximal CpG sites mostly occur in clusters throughout the genome, we will concentrate on the changes of DMRs. The Venn diagram showed that 364, 391, 442, and 517 unique gene from DMRs between Con and Ost were exclusively observed at day 1, 7, 14, and 21, respectively (Figure 1f). In addition, there were 491, 512, 502, 635, and 534 unique genes in DMRs between AS and DL at day 0, 1, 7, 14, and 21 (Figure 1g). The full list of DMRs in different comparisons was shown in Supplementary Table S3.

**Figure 2.**
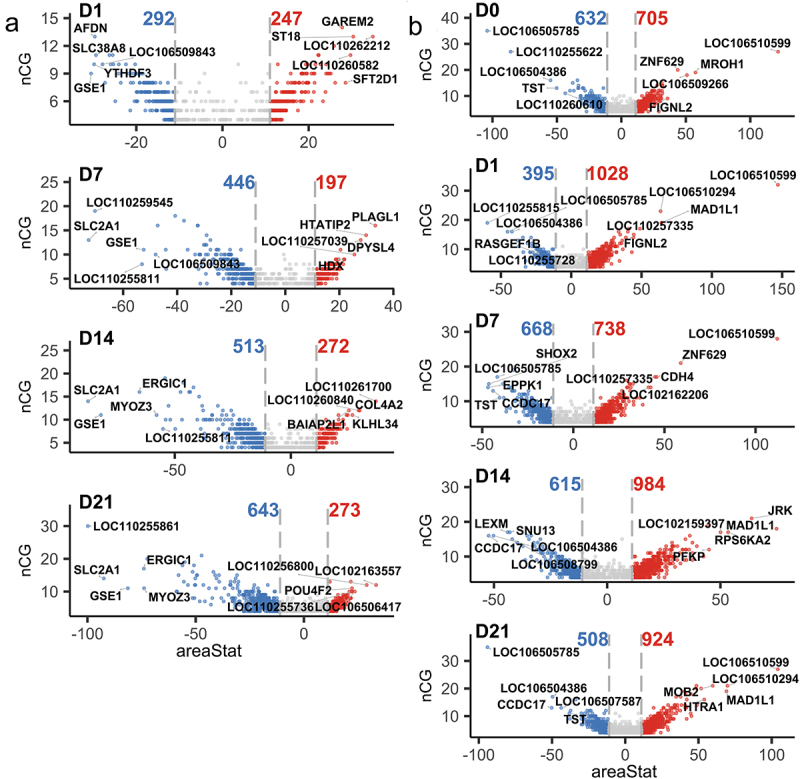
(a) Volcano plots of DMRs between control SMSCs and osteogenic induced SMSCs at each time point. (b) Volcano plots of DMRs between AS and DL prior to and after osteogenic differentiation at each time point.

Differential methylation analysis revealed that the number of DMRs between osteogenic-induced SMSCs and undifferentiated SMSCs consistently increased from 539 on day 1 to 916 on day 21, mainly due to the increase in the number of hypomethylated DMRs in differentiating SMSCs from 292 on day 1 to 643 on day 21 (Figure 2a). Approximately two-thirds of DMRs showed hypomethylation in differentiating SMSCs between day 7 and day 21 relative to undifferentiated SMSCs (Figure 2a). Interestingly, in differentiating cells, the DMRs-related genes *GSE1* and *SLC2A1* were among the top 5 hypomethylated DMRs at most time points (Figure 2a). Comparing AS and DL, the number of DMRs (around 1400) remains stable during osteogenic differentiation, except for a slight peak at day 14. The SMSCs isolated from AS pigs always had a higher number of hypermethylated DMRs than those from DL pigs, especially at day 1 and day 21 (Figure 2b), which is consistent with the result of a higher global median methylation level in AS. The most common DMRs found at different times were *TST, LOC106505785* and *LOC106510599* (Figure 2b).

## Functional enrichment of DMRs

**Figure 3.**
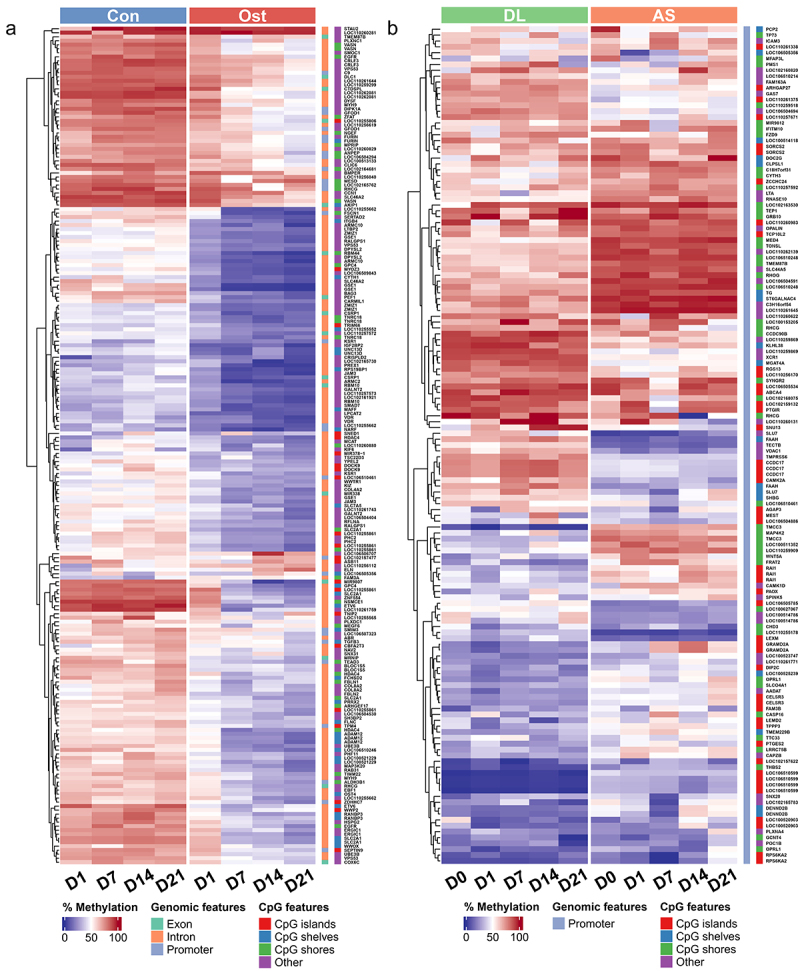
(a) Methylation heatmap of 209 DMRs with relative methylation difference ≥ 30% in the promoter or gene body regions between control SMSCs and osteogenic induced SMSCs at each time point. (b) Methylation heatmap of 143 DMRs with relative methylation difference ≥ 30% in the promoter region between AS and DL prior to and after osteogenic differentiation at each time point.

**Figure 4.**
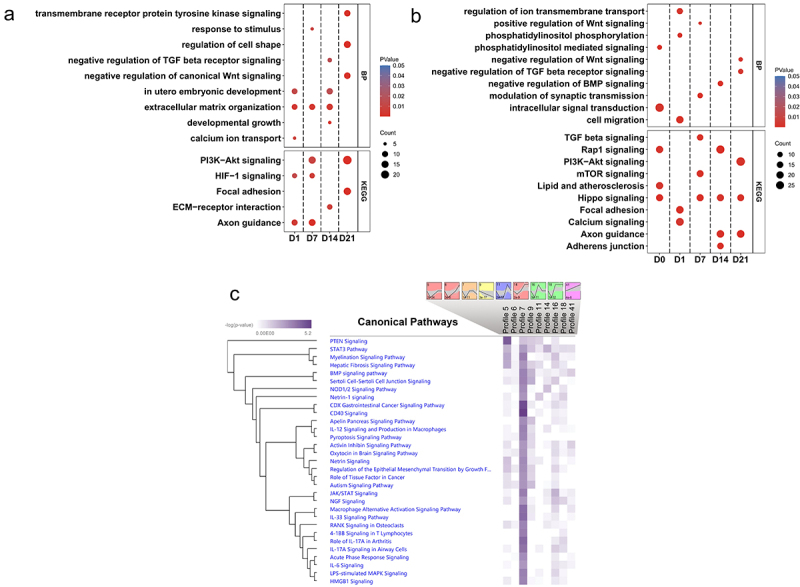
(a) BP and KEGG pathway enrichment analysis of DMRs-associated genes between control SMSCs and osteogenic induced SMSCs at each time point. (b) BP and KEGG pathway enrichment analysis of DMRs-associated genes between AS and DL prior to and after osteogenic differentiation at each time point. (c) IPA canonical pathway analysis of DMRs-associated genes from 9 significant time-series profiles detected by STEM.

The complete results of functional enrichment analysis can be found in Supplementary Table S4. The hierarchical clustering heatmap showed that methylation differences between the state of SMSCs (Figure 3a) and between the different breeds (Figure 3b). During osteogenic induction of SMSCs, both Axon guidance and HIF-1 signalling pathway were enriched at the early stage of osteogenic induction, while extracellular matrix organization was observed from day 1 to day 14 (Figure 4a). Two pathways engaging with extracellular matrix, ECM-receptor interaction and Focal adhesion, were also found at day 14 and day 21, respectively (Figure 4a). To be specific, hypomethylated DMRs associated with extracellular matrix during osteogenic induction were functionally assigned, for instance, *FBLN1, ITGB4, HSPG2*, and *ELN* (Figure 3a, Supplementary Table S5). Other pathways that have universal impact on cell growth and differentiation were observed at the late stage of osteogenic induction, such as developmental growth, PI3K-AKt signalling, and transmembrane receptor protein tyrosine kinase signalling pathway (Figure 4a). Furthermore, several pathways that are widely perceived as contributing to osteogenic differentiation were also identified in this study, including Wnt, BMP, and TGFB signalling pathways. Most DMRs in these pathways were also hypomethylated in osteogenic SMSCs compared to undifferentiated SMSCs, covering genes such as, *WWTR1, SMAD7, BMPER*, and *TGFB3* (Figure 3a, Supplementary Table S4).

When comparing osteogenic SMSCs isolated from AS and DL, the Hippo pathway was observed throughout the induction period, except on day 1 (Figure 4b). The lipid and phospholipid pathways, namely lipid and atherosclerosis, phosphatidylinositol-mediated signalling and phosphatidylinositol phosphorylation, were enriched at day 0 and/or day 1. As osteogenesis progressed, the TGFB, Wnt and BMP signalling pathways were enriched at mid and late stages of induction. The hierarchical clustering heatmap showed that the methylation differences between AS and DL in many promoter DMRs persisted over all time points (Figure 3b).

## Temporal changes in DNA methylation along osteogenic induction

We observed 9 significant time-varying methylation profiles, with profiles 9 and 41 showing the patterns of progressive decrease and increase of methylation, respectively, over time compared to the control (day 0) (Figure 4c). Profile 7 is enriched for genes associated with most canonical pathways, including CD40, IL-17A in arthritis, BMP, RANK pathways, etc (Figure 4c).

## Integration of DNA methylation and gene expression

**Figure 5.**
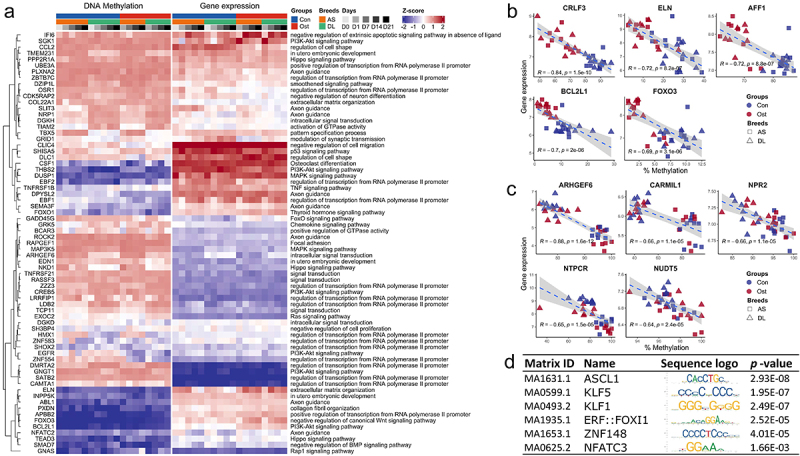
(a) Genes shown negative correlation between DNA methylation and gene expression and its enriched pathways. (b) Top 5 negative correlations between methylation levels and gene expression in the comparisons of control SMSCs and osteogenic induced SMSCs. (c) Top 5 negative correlations between methylation levels and gene expression in the comparisons of SMSCs from as and DL. (d) Most frequently enriched TF binding motifs in promoter DMRs.

To identify potential methylation-driven genes associated with osteogenic induction, we used previously collected microarray data from the same sample set. In total, we found 340 DMRs whose associated genes showed a negative correlation between DNA methylation level and transcript abundance, with 156 and 183 genes in the differentiation state (Ost vs. Con) and breed (AS vs. DL) comparisons, respectively (Supplementary Table S5). The heatmap of genes belonging to different biological processes or pathways illustrates the relationships between the expression and methylation patterns (Figure 5a). The top 5 genes in terms of stringency of correlation between methylation and expression, located in promoter or gene body regions, were *CRLF3, ELN, AFF1, BCL2L1* and F*OXO3*, most of which were hypomethylated in osteogenic SMSCs compared to undifferentiated SMSCs at the late stage of induction (Figure 5b, Supplementary Table S6). When comparing AS and DL, the top candidates were *ARHGEF6, CARMIL1, NPR2, NTPCR* and *NUDT5*, of which *CARMIL1* consistently showed higher methylation than DL in AS at all time points (Figure 5c, Supplementary Table S5).

## DNA methylation patterns within TFBS

To better understand the regulatory potential of DMRs during osteogenic induction, we searched for consensus sequences representing transcription factor binding sites within the promoter DMRs. Comparison of cell differentiation states revealed 25 promoter DMRs in which TFBSs of 68 non-redundant transcription factors were mapped (Supplementary Table S6). The transcription factors that were enriched more than fivefold were *KLF1, NFATC3, ZNF148, ASCL1*, *ERF:FOXI1* and *KLF5* (Figure 5c). In the breed comparisons, 138 promoter DMRs were found in which TFBSs of 105 non-redundant transcription factors were enriched, of which *NFATC3, KLF5* and *ZNF148* were the most abundant (Figure 5d).

## Discussion

We have previously investigated dynamic changes in the transcriptome profile of SMSCs derived from two different genetic backgrounds of porcine breeds during in vitro osteogenesis [4]. In addition to gene expression, DNA methylation, a mechanism of epigenetic regulation, is also associated with cell lineage commitment, including osteogenic differentiation [15,38]. Therefore, in this study, we provide further comprehensive insights into the dynamic changes in DNA methylation patterns related to osteogenic differentiation and the impact of distinct metabolic donor types on porcine SMSCs.

Regarding the global methylation pattern, our study showed that the majority of CpGs had high methylation levels, except those located in promoter regions or CpG islands, which is consistent with many previous studies [5]. Previous studies have shown that DNA methylation dynamics differ in haematopoietic stem cells derived from different sources and cell type differentiation [39,40]. They found changes in DNA methylation associated with commitment to the myeloid and lymphoid lineages [40]. In our study, we found that the origin of donor cells in terms of the breed used had a significant effect on DNA methylation changes, while a lesser change in DNA methylation was linked to a commitment to the osteogenic process.

Most DMRs were hypomethylated in osteogenic SMSCs compared to undifferentiated SMSCs, as found in this study. This reduction in DNA methylation levels coincided with lower expression levels of three key DNA methyltransferases (DNMTs) - *DNMT1, DNMT3a*, and *DNMT3b*, particularly in the late stage of osteogenic induction. Since these enzymes are crucial for establishing and maintaining DNA methylation patterns [5], it appears that the low methylation of specific genes contributes to the osteogenic transition of SMSCs. Additionally, numerous studies have reported that DNA methylation inhibitors could enhance the osteogenic capability of MSCs [8,41], and even improve their potential under unfavourable circumstances [42,43].

Similar to our earlier study in gene expression [4], we observed extracellular matrix related pathways throughout the entire induction period. The extracellular matrix is mainly produced by osteoblast before mineralization and could enhance osteogenic differentiation of MSCs [44]. ELN (Elastin) makes up part of the extracellular matrix and regulates MSCs proliferation and differentiation through its biological and mechanical properties [45]. A significant negative correlation between gene expression and methylation level of ELN has been found from day 7 to day 21 of differentiation in this study, suggesting the methylation pattern of ELN may influence its expression during osteogenic induction. Additionally, it has also been assumed that the level of ELN may act as a predictive indicator for the osteogenic ability of MSCs [46].

The commitment of MSCs to the osteogenic lineage requires the involvement of BMP and TGF-β signalling pathways [47,48]. In this study, we noticed that the *BMPER*, encoding a secreted protein that interacts with BMP functions, was hypermethylated on days 1 and 7. However, due to the biphasic effects of *BMPER* on BMPs [49,50], its exact role in osteogenic differentiation remains to be investigated. In addition, *TGFB3* showed a hypomethylation pattern during the late stage of induction, whereby *TGFB3* mRNA levels were upregulated in osteogenic-induced SMSCs at day 21 [4]. It has been reported that the overexpression of TGFB3 contributes to the osteogenic differentiation of MSCs [51]. Interestingly, *SMAD7*, an antagonist of TGF-β signalling [52], was hypomethylated in our study and showed negatively correlated gene expression. A repressive effect of SMAD7 on the differentiation and mineralization of osteoblasts has been observed [53,54]. On the other hand, SMAD7 knockout mice exhibited impaired ossification [55]. A recent study showed that SMAD7 knockdown in human SMSCs enhanced chondrogenic differentiation and suppressed endochondral ossification [56]. Our study further substantiates the role of *SMAD7* during osteogenic and chondrogenic differentiation.

We also identified several upregulated genes, possibly driven by their hypomethylation pattern during the mid and late stages of osteogenic induction, such as *CRLF3, AFF1, BCL2L1*, and *FOXO3*. CRLF3 could be associated with neuronal differentiation [57], but its function in osteogenesis is currently unknown. AFF1 was regarded as a negative regulator in osteogenic differentiation [58]. Unexpectedly, our results seem to indicate a positive effect of *AFF1* on osteogenic differentiation driven by methylation changes. In addition, the results for two other genes, *BCL2L1* and *FOXO3*, are consistent with the observation that *BCLXL* and *FOXO3* contribute to osteogenic differentiation [59,60]. Further experiments are required to explore the detailed mechanism of DNA methylation in these genes across osteogenesis.

A growing body of evidence showed that metabolic characteristics of donors, for example age and obesity, affect the methylation profile of their MSCs [61,62]. Such differences also occurred in the MSCs of pigs induced by high-fat diet [26,27]. It has also been demonstrated that obesity or metabolic syndrome could impede the osteogenic differentiation of MSCs [19,21,22]. However, it is unclear how the epigenetic changes affect the osteogenic differentiation of MSCs from donors with distinct metabolic phenotypes. DL is a modern pig breed with high lean meat, whereas AS is featured by fatness. Thus, we employed SMSCs from these two breeds to identify DNA methylation changes along with osteogenic induction. Characterization of global methylation shows an increased level of methylation in SMSCs from AS when compared to DL, consistent with that observed in skeletal muscle and similar tissues from obese and lean pig breeds [63,64], while this may be connected with the higher expression level of *DNMT1*, observed in this study. As a modern breed, DL has been selected for efficiency, lean meat content and commercial purposes, whereas AS has been bred in recent centuries to maintain its native characteristics. Epigenetic modifications have also contributed to breed differentiation, as shown by the comparison of non-induced SMSCs. Selective breeding strategies in the commercial breed (DL) and conservation breeding in the ancient breed (AS) have resulted in the emergence of new alleles and new epialleles. The resulting genetic differentiation affects multiple metabolic and signalling pathways, including developmental patterns. The induction of osteogenesis involves a specific spectrum of genes and molecular pathways, some of which are breed-specific and mediated by epigenetic differences, but there is also a high degree of overlap between breeds.

The osteogenesis-related signalling pathways, including TGF-beta, Wnt, and BMP, were observed exhibiting differentially methylated genes at various time points, suggesting that methylation regulates the osteogenic processes of SMSCs from AS and DL. The most frequently enriched pathway was Hippo signalling in the comparison of AS and DL. However, there was no significant change in the methylation pattern of core components in Hippo signalling [65]. On the other hand, several DMRs enriched in Hippo signalling were also key effectors of WNT signalling, for instance, *WNT5A* and *TCF7L2*. These two high-variable DMRs were hypermethylated in AS compared to DL. Experiments have shown that hypermethylation of WNT5A negatively regulated the function of limbal epithelial stem cells from diabetic donors [66]. It has also been demonstrated that the genetic variation of *TCF7L2* is highly correlated with obese traits in pigs and type-2 diabetes in humans [67,68]. At the same time, both *WNT5A* and *TCF7L2* were regarded as important regulators of osteoblast formation lately [69,70]. Regarding the identification of possible methylation-driven genes, there was very little evidence about the role of these genes detected in this study with regard to pig breeds or osteogenic differentiation. DNA methylation could directly impede the binding of transcription factors to their respective sites, thereby controlling gene expression [71]. Hence, we examined the TFBS motifs enriched in promoter DMRs. The sequence motif of two members from the krüppel-like factor (KLF) family, *KLF1* and *KLF5*, were detected. KLFs play diverse roles in various cellular processes, including skeletal development and pathologies [72,73]. KLF1 is a vital transcription factor in erythropoiesis, yet there is no literature regarding its function in bone development [72]. KLF5 is expressed in osteoblasts and may mediate endochondral ossification [74], while knockdown of KLF5 inhibited osteogenic differentiation [75]. Moreover, NFATC3, a downstream regulator of NFATC1, was thought to maintain bone homoeostasis through the regulation of RANKL expression [76].

## Conclusions

In this study, we found that the most DMRs were hypomethylated in osteogenic SMSCs compared to undifferentiated SMSCs. These findings indicate that the demethylation of specific genes plays an important role in the osteogenic transition process. In addition, we demonstrated the persistence of the breed-specific epigenetic pattern and the acquisition of an osteogenic phenotype in cell culture, while maintaining donor memory. It provides evidence that the differences in osteogenic differentiation depending on the breed origin of the donor tissue are partly mediated by epigenetic differences, which, in addition to genetic differences, are associated with breed-specific differences in metabolic type. Overall, the DNA methylation pattern of SMSCs from AS and DL pigs retains some inherent features during osteogenic induction that may confer a distinct osteogenic capacity.

## Supplementary Material

Subplementary Table_revised.xlsx

## Data Availability

All RRBS sequencing data have been deposited in the ArrayExpress database at EMBL-EBI (www.ebi.ac.uk/arrayexpress) under the accession number E-MTAB-9913.

## References

[1] Mochizuki T, Muneta T, Sakaguchi Y, et al. Higher chondrogenic potential of fibrous synovium– and adipose synovium–derived cells compared with subcutaneous fat–derived cells: Distinguishing properties of mesenchymal stem cells in humans. Arthritis Rheum. 2006;54(3):843–15. doi: 10.1002/art.21651 16508965

[2] Bami M, Sarlikiotis T, Milonaki M, et al. Superiority of synovial membrane mesenchymal stem cells in chondrogenesis, osteogenesis, myogenesis and tenogenesis in a rabbit model. Injury. 2020;51(12):2855–2865. doi: 10.1016/j.injury.2020.03.022 32201117

[3] Li N, Gao J, Mi L, et al. Synovial membrane mesenchymal stem cells: past life, current situation, and application in bone and joint diseases. Stem Cell Res Ther. 2020;11(1):381. doi: 10.1186/s13287-020-01885-3 32894205 PMC7487958

[4] Li S, Siengdee P, Oster M, et al. Transcriptome changes during osteogenesis of porcine mesenchymal stem cells derived from different types of synovial membranes and genetic background. Sci Rep. 2023;13(1):10048. doi: 10.1038/s41598-023-37260-4 37344635 PMC10284927

[5] Greenberg MVC, Bourc’his D. The diverse roles of DNA methylation in mammalian development and disease. Nat Rev Mol Cell Biol. 2019;20(10):590–607. doi: 10.1038/s41580-019-0159-6 31399642

[6] Montecino M, Carrasco ME, Nardocci G. Epigenetic control of osteogenic lineage commitment. Front Cell Dev Biol. 2020;8:611197. doi: 10.3389/fcell.2020.611197 33490076 PMC7820369

[7] Wang X, Yu F, Ye L. Epigenetic control of mesenchymal stem cells orchestrates bone regeneration. Front Endocrinol (Lausanne). 2023;14:1126787. doi: 10.3389/fendo.2023.1126787 36950693 PMC10025550

[8] El-Serafi AT, Oreffo RO, Roach HI. Epigenetic modifiers influence lineage commitment of human bone marrow stromal cells: Differential effects of 5-aza-deoxycytidine and trichostatin A. Differentiation. 2011;81(1):35–41. doi: 10.1016/j.diff.2010.09.183 20970916

[9] Alghfeli L, Parambath D, Tag Eldeen LA, et al. Non-additive effect of the DNA methylation inhibitor, 5-Aza-dC, and glass as a culture surface on osteogenic differentiation. Heliyon. 2022;8(12):e12433. doi: 10.1016/j.heliyon.2022.e12433 36590514 PMC9794900

[10] Yin Y, Morgunova E, Jolma A, et al. Impact of cytosine methylation on DNA binding specificities of human transcription factors. Science. 2017;356(6337):356. doi: 10.1126/science.aaj2239 28473536 PMC8009048

[11] Daniunaite K, Serenaite I, Misgirdaite R, et al. Epigenetic regulation of human adipose-derived stem cells differentiation. Mol Cell Biochem. 2015;410(1–2):111–120. doi: 10.1007/s11010-015-2543-7 26307369

[12] Marofi F, Hassanzadeh A, Solali S, et al. Epigenetic mechanisms are behind the regulation of the key genes associated with the osteoblastic differentiation of the mesenchymal stem cells: The role of zoledronic acid on tuning the epigenetic changes. J Cell Physiol. 2019;234(9):15108–15122. doi: 10.1002/jcp.28152 30652308

[13] Wang Z, Wen S, Zhong M, et al. Epigenetics: Novel crucial approach for osteogenesis of mesenchymal stem cells. J Tissue Eng. 2023;14:20417314231175364. doi: 10.1177/20417314231175364 37342486 PMC10278427

[14] Luo C, Hajkova P, Ecker JR. Dynamic DNA methylation: In the right place at the right time. Science. 2018;361(6409):1336–1340. doi: 10.1126/science.aat6806 30262495 PMC6197482

[15] Yu F, Shen H, Deng HW. Systemic analysis of osteoblast-specific DNA methylation marks reveals novel epigenetic basis of osteoblast differentiation. Bone Rep. 2017;6:109–119. doi: 10.1016/j.bonr.2017.04.001 28409176 PMC5384298

[16] Ferreira RS, Assis RIF, Feltran GDS, et al. Genome-wide DNA (hydroxy) methylation reveals the individual epigenetic landscape importance on osteogenic phenotype acquisition in periodontal ligament cells. J Periodontol. 2022;93(3):435–448. doi: 10.1002/JPER.21-0218 34291826

[17] Gomez R, Barter MJ, Alonso-Perez A, et al. DNA methylation analysis identifies key transcription factors involved in mesenchymal stem cell osteogenic differentiation. Biol Res. 2023;56(1):9. doi: 10.1186/s40659-023-00417-6 36890579 PMC9996951

[18] van Gastel N, Carmeliet G. Metabolic regulation of skeletal cell fate and function in physiology and disease. Nat Metab. 2021;3(1):11–20. doi: 10.1038/s42255-020-00321-3 33398192

[19] Frazier TP, Gimble JM, Devay JW, et al. Body mass index affects proliferation and osteogenic differentiation of human subcutaneous adipose tissue-derived stem cells. BMC Cell Biol. 2013;14(1):34. doi: 10.1186/1471-2121-14-34 23924189 PMC3750383

[20] De Girolamo L, Stanco D, Salvatori L, et al. Stemness and osteogenic and adipogenic potential are differently impaired in subcutaneous and visceral adipose derived stem cells (ASCs) isolated from obese donors. Int J Immunopathol Pharmacol. 2013;26:11–21. doi: 10.1177/03946320130260S103 24046945

[21] Oliva-Olivera W, Leiva Gea A, Lhamyani S, et al. Differences in the osteogenic differentiation capacity of omental adipose-derived stem cells in obese patients with and without metabolic syndrome. Endocrinology. 2015;156(12):4492–4501. doi: 10.1210/en.2015-1413 26372179 PMC4655209

[22] Strong AL, Hunter RS, Jones RB, et al. Obesity inhibits the osteogenic differentiation of human adipose-derived stem cells. J Transl Med. 2016;14(1):27. doi: 10.1186/s12967-016-0776-1 26818763 PMC4730660

[23] Luthje FL, Skovgaard K, Jensen HE, et al. Pigs are useful for the molecular study of bone inflammation and regeneration in humans. Lab Anim. 2018;52(6):630–640. doi: 10.1177/0023677218766391 29653496

[24] Hotham WE, Henson FMD. The use of large animals to facilitate the process of MSC going from laboratory to patient—‘bench to bedside’. Cell Biol Toxicol. 2020;36(2):103–114. doi: 10.1007/s10565-020-09521-9 32206986 PMC7196082

[25] Afarideh M, Thaler R, Khani F, et al. Global epigenetic alterations of mesenchymal stem cells in obesity: the role of vitamin C reprogramming. Epigenetics. 2021;16(7):705–717. doi: 10.1080/15592294.2020.1819663 32893712 PMC8216191

[26] Glasstetter LM, Oderinde TS, Mirchandani M, et al. Obesity and dyslipidemia are associated with partially reversible modifications to DNA hydroxymethylation of apoptosis- and senescence-related genes in swine adipose-derived mesenchymal stem/stromal cells. Stem Cell Res Ther. 2023;14(1):143. doi: 10.1186/s13287-023-03372-x 37231414 PMC10214739

[27] Rajagopalan KS, Kazeminia S, Glasstetter LM, et al. Metabolic syndrome induces epigenetic alterations in mitochondria-related genes in swine mesenchymal stem cells. Cells. 2023;12(9):12. doi: 10.3390/cells12091274 PMC10177475 37174674

[28] Siengdee P, Oster M, Reyer H, et al. Morphological and Molecular Features of Porcine Mesenchymal Stem Cells Derived from Different Types of Synovial Membrane, and Genetic Background of Cell Donors. Front Cell Dev Biol. 2020;8:8. doi: 10.3389/fcell.2020.601212 33363158 PMC7755640

[29] Ponsuksili S, Trakooljul N, Basavaraj S, et al. Epigenome-wide skeletal muscle DNA methylation profiles at the background of distinct metabolic types and ryanodine receptor variation in pigs. BMC Genomics. 2019;20(1):20. doi: 10.1186/s12864-019-5880-1 31195974 PMC6567458

[30] Park Y, Wu H. Differential methylation analysis for BS-seq data under general experimental design. Bioinformatics. 2016;32(10):1446–1453. doi: 10.1093/bioinformatics/btw026 26819470 PMC12157722

[31] Law CW, Zeglinski K, Dong X, et al. A guide to creating design matrices for gene expression experiments. F1000Res. 2020;9:1444. doi: 10.12688/f1000research.27893.1 33604029 PMC7873980

[32] Wang Q, Li M, Wu T, et al. Exploring Epigenomic Datasets by ChIPseeker. Curr Protoc. 2022;2(10):e585. doi: 10.1002/cpz1.585 36286622

[33] Gu Z. Complex heatmap visualization. iMeta. 2022;1(3):e43. doi: 10.1002/imt2.43 38868715 PMC10989952

[34] Ernst J, Bar-Joseph Z. STEM: a tool for the analysis of short time series gene expression data. BMC Bioinformatics. 2006;7(1):191. doi: 10.1186/1471-2105-7-191 16597342 PMC1456994

[35] Castro-Mondragon JA, Riudavets-Puig R, Rauluseviciute I, et al. JASPAR 2022: the 9th release of the open-access database of transcription factor binding profiles. Nucleic Acids Res. 2022;50(D1):D165–D73. doi: 10.1093/nar/gkab1113 34850907 PMC8728201

[36] Tan G, Lenhard B. TFBSTools: an R/bioconductor package for transcription factor binding site analysis. Bioinformatics. 2016;32(10):1555–1556. doi: 10.1093/bioinformatics/btw024 26794315 PMC4866524

[37] McLeay RC, Bailey TL. Motif enrichment analysis: a unified framework and an evaluation on ChIP data. BMC Bioinformatics. 2010;11(1):165. doi: 10.1186/1471-2105-11-165 20356413 PMC2868005

[38] Suelves M, Carrio E, Nunez-Alvarez Y, et al. DNA methylation dynamics in cellular commitment and differentiation. Brief Funct Genomics. 2016;15:443–453. doi: 10.1093/bfgp/elw017 27416614

[39] Laurent L, Wong E, Li G, et al. Dynamic changes in the human methylome during differentiation. Genome Res. 2010;20(3):320–331. doi: 10.1101/gr.101907.109 20133333 PMC2840979

[40] Farlik M, Halbritter F, Muller F, et al. DNA methylation dynamics of human hematopoietic stem cell differentiation. Cell Stem Cell. 2016;19(6):808–822. doi: 10.1016/j.stem.2016.10.019 27867036 PMC5145815

[41] Zhou GS, Zhang XL, Wu JP, et al. 5-Azacytidine facilitates osteogenic gene expression and differentiation of mesenchymal stem cells by alteration in DNA methylation. Cytotechnology. 2009;60(1–3):11. doi: 10.1007/s10616-009-9203-2 19557538 PMC2780539

[42] Yan X, Ehnert S, Culmes M. 5-azacytidine improves the osteogenic differentiation potential of aged human adipose-derived mesenchymal stem cells by DNA demethylation. PLOS ONE. 2014;9(3):e90846. doi: 10.1371/journal.pone.0090846 24603866 PMC3946260

[43] Liu Z, Chen T, Sun W, et al. DNA demethylation rescues the impaired osteogenic differentiation ability of human periodontal ligament stem cells in high glucose. Sci Rep. 2016;6(1):27447. doi: 10.1038/srep27447 27273319 PMC4897703

[44] Mansour A, Mezour MA, Badran Z, et al. Extracellular Matrices for Bone Regeneration: A literature review. Tissue Eng Part A. 2017;23(23–24):1436–1451. doi: 10.1089/ten.TEA.2017.0026 28562183

[45] Lee S, Kim JE, Seo HJ, et al. Design of fibronectin type III domains fused to an elastin-like polypeptide for the osteogenic differentiation of human mesenchymal stem cells. Acta Biochim Biophys Sin (Shanghai). 2019;51(8):856–863. doi: 10.1093/abbs/gmz063 31267123

[46] Twine NA, Chen L, Pang CN, et al. Identification of differentiation-stage specific markers that define the ex vivo osteoblastic phenotype. Bone. 2014;67:23–32. doi: 10.1016/j.bone.2014.06.027 24984278

[47] Wu M, Chen G, Li YP. TGF-β and BMP signaling in osteoblast, skeletal development, and bone formation, homeostasis and disease. Bone Res. 2016;4(1):16009. doi: 10.1038/boneres.2016.9 27563484 PMC4985055

[48] Grafe I, Alexander S, Peterson JR, et al. TGF-β Family signaling in mesenchymal differentiation. Cold Spring Harb Perspect Biol. 2018;10(5):10. doi: 10.1101/cshperspect.a022202 PMC5932590 28507020

[49] Serpe M, Umulis D, Ralston A, et al. The BMP-binding protein Crossveinless 2 is a short-range, concentration-dependent, biphasic modulator of BMP signaling in Drosophila. Dev Cell. 2008;14(6):940–953. doi: 10.1016/j.devcel.2008.03.023 18539121 PMC2488203

[50] Satomi-Kobayashi S, Kinugasa M, Kobayashi R, et al. Osteoblast-like differentiation of cultured human coronary artery smooth muscle cells by bone morphogenetic protein endothelial cell precursor-derived regulator (BMPER). J Biol Chem. 2012;287(36):30336–30345. doi: 10.1074/jbc.M111.329110 22778264 PMC3436285

[51] He W, Chen L, Huang Y, et al. Synergistic effects of recombinant Lentiviral-mediated BMP2 and TGF-beta3 on the osteogenic differentiation of rat bone marrow mesenchymal stem cells in vitro. Cytokine. 2019;120:1–8. doi: 10.1016/j.cyto.2019.03.020 30991228

[52] Yan X, Liao H, Cheng M, et al. Smad7 protein interacts with receptor-regulated smads (R-Smads) to inhibit transforming growth factor-β (TGF-β)/Smad signaling. J Biol Chem. 2016;291(1):382–392. doi: 10.1074/jbc.M115.694281 26555259 PMC4697173

[53] Yano M, Inoue Y, Tobimatsu T, et al. Smad7 inhibits differentiation and mineralization of mouse osteoblastic cells. Endocr J. 2012;59(8):653–662. doi: 10.1507/endocrj.ej12-0022 22673292

[54] Vishal M, Vimalraj S, Ajeetha R, et al. MicroRNA-590-5p Stabilizes Runx2 by Targeting Smad7 During Osteoblast Differentiation. J Cell Physiol. 2017;232(2):371–380. doi: 10.1002/jcp.25434 27192628

[55] Estrada KD, Wang W, Retting KN, et al. Smad7 regulates terminal maturation of chondrocytes in the growth plate. Dev Biol. 2013;382(2):375–384. doi: 10.1016/j.ydbio.2013.08.021 23994637 PMC4267888

[56] Xiao P, Zhu Z, Du C, et al. Silencing Smad7 potentiates BMP2-induced chondrogenic differentiation and inhibits endochondral ossification in human synovial-derived mesenchymal stromal cells. Stem Cell Res Ther. 2021;12(1):132. doi: 10.1186/s13287-021-02202-2 33588941 PMC7885459

[57] Wegscheid ML, Anastasaki C, Hartigan KA, et al. Patient-derived iPSC-cerebral organoid modeling of the 17q11.2 microdeletion syndrome establishes CRLF3 as a critical regulator of neurogenesis. Cell Rep. 2021;36(1):109315. doi: 10.1016/j.celrep.2021.109315 34233200 PMC8278229

[58] Zhou CC, Xiong QC, Zhu XX, et al. AFF1 and AFF4 differentially regulate the osteogenic differentiation of human MSCs. Bone Res. 2017;5(1):17044. doi: 10.1038/boneres.2017.44 28955517 PMC5613922

[59] Ambrogini E, Almeida M, Martin-Millan M, et al. FoxO-mediated defense against oxidative stress in osteoblasts is indispensable for skeletal homeostasis in mice. Cell Metab. 2010;11(2):136–146. doi: 10.1016/j.cmet.2009.12.009 20142101 PMC2819984

[60] Moriishi T, Fukuyama R, Miyazaki T, et al. Overexpression of BCLXL in osteoblasts inhibits osteoblast apoptosis and increases bone volume and strength. J Bone Miner Res. 2016;31(7):1366–1380. doi: 10.1002/jbmr.2808 26852895

[61] Pasumarthy KK, Doni Jayavelu N, Kilpinen L, et al. Methylome analysis of human bone marrow MSCs reveals extensive age- and culture-induced changes at distal regulatory elements. Stem Cell Reports. 2017;9(3):999–1015. doi: 10.1016/j.stemcr.2017.07.018 28844656 PMC5599244

[62] Xie S, Choudhari S, Wu CL, et al. Aging and obesity prime the methylome and transcriptome of adipose stem cells for disease and dysfunction. Faseb J. 2023;37(3):e22785. doi: 10.1096/fj.202201413R 36794668 PMC10561192

[63] Yang Y, Zhou R, Mu Y, et al. Genome-wide analysis of DNA methylation in obese, lean, and miniature pig breeds. Sci Rep. 2016;6(1):30160. doi: 10.1038/srep30160 27444743 PMC4957084

[64] Yi G, Liu L, Yao Y, et al. Multi-omics analysis reveals signatures of selection and loci associated with complex traits in pigs. bioRxiv. doi: 10.1101/2023.09.19.558553 PMC12625778 41262543

[65] Pan JX, Xiong L, Zhao K, et al. YAP promotes osteogenesis and suppresses adipogenic differentiation by regulating β-catenin signaling. Bone Res. 2018;6(1):18. doi: 10.1038/s41413-018-0018-7 29872550 PMC5984632

[66] Shah R, Spektor TM, Weisenberger DJ, et al. Reversal of dual epigenetic repression of non-canonical Wnt-5a normalises diabetic corneal epithelial wound healing and stem cells. Diabetologia. 2023;66(10):1943–1958. doi: 10.1007/s00125-023-05960-1 37460827 PMC10474199

[67] Hattersley AT. Prime suspect: the TCF7L2 gene and type 2 diabetes risk. J Clin Invest. 2007;117(8):2077–2079. doi: 10.1172/JCI33077 17671643 PMC1934573

[68] Du ZQ, Fan B, Zhao X, et al. Association analyses between type 2 diabetes genes and obesity traits in pigs. Obesity (Silver Spring). 2009;17(2):323–329. doi: 10.1038/oby.2008.557 19057525

[69] Maeda K, Kobayashi Y, Udagawa N, et al. Wnt5a-Ror2 signaling between osteoblast-lineage cells and osteoclast precursors enhances osteoclastogenesis. Nat Med. 2012;18(3):405–412. doi: 10.1038/nm.2653 22344299

[70] Mohan S, Kesavan C. T-cell factor 7L2 is a novel regulator of osteoblast functions that acts in part by modulation of hypoxia signaling. Am J Physiol Endocrinol Metab. 2022;322(6):E528–E39. doi: 10.1152/ajpendo.00035.2022 35466691 PMC9169825

[71] Kaluscha S, Domcke S, Wirbelauer C, et al. Evidence that direct inhibition of transcription factor binding is the prevailing mode of gene and repeat repression by DNA methylation. Nat Genet. 2022;54(12):1895–1906. doi: 10.1038/s41588-022-01241-6 36471082 PMC9729108

[72] Abe M, Saeki N, Ikeda Y, et al. Kruppel-like Factors in Skeletal Physiology and Pathologies. Int J Mol Sci. 2022;23(23):15174. doi: 10.3390/ijms232315174 36499521 PMC9741390

[73] Zakeri S, Aminian H, Sadeghi S, et al. Kruppel-like factors in bone biology. Cell Signal. 2022;93:110308. doi: 10.1016/j.cellsig.2022.110308 35301064

[74] Shinoda Y, Ogata N, Higashikawa A, et al. Krüppel-like factor 5 causes cartilage degradation through transactivation of matrix metalloproteinase 9. J Biol Chem. 2008;283(36):24682–24689. doi: 10.1074/jbc.M709857200 18617520 PMC3259811

[75] Wangzhou K, Lai Z, Lu Z, et al. MiR-143-3p Inhibits osteogenic differentiation of human periodontal ligament cells by targeting KLF5 and inactivating the Wnt/beta-catenin pathway. Front Physiol. 2020;11:606967. doi: 10.3389/fphys.2020.606967 33603676 PMC7884451

[76] Lee HL, Bae OY, Baek KH, et al. High extracellular calcium-induced NFATc3 regulates the expression of receptor activator of NF-κB ligand in osteoblasts. Bone. 2011;49(2):242–249. doi: 10.1016/j.bone.2011.04.006 21514407

